# ecTMB: a robust method to estimate and classify tumor mutational burden

**DOI:** 10.1038/s41598-020-61575-1

**Published:** 2020-03-18

**Authors:** Lijing Yao, Yao Fu, Marghoob Mohiyuddin, Hugo Y. K. Lam

**Affiliations:** Roche Sequencing Solutions, Santa Clara, CA 95050 USA

**Keywords:** Computational models, Cancer

## Abstract

Tumor Mutational Burden (TMB) is a measure of the abundance of somatic mutations in a tumor, which has been shown to be an emerging biomarker for both anti-PD-(L)1 treatment and prognosis; however, multiple challenges still hinder the adoption of TMB as a biomarker. The key challenges are the inconsistency of tumor mutational burden measurement among assays and the lack of a meaningful threshold for TMB classification. Here we describe a new method, ecTMB (Estimation and Classification of TMB), which uses an explicit background mutation model to predict TMB robustly and to classify samples into biologically meaningful subtypes defined by tumor mutational burden.

## Introduction

Cancers can be caused by an accumulation of genetic mutations in oncogenes or tumor suppressors^1^. These mutations are known as “driver” mutations and they are under positive selection; however, only a very small fraction of somatic mutations in a tumor sample are expected to be drivers. The remaining majority of somatic mutations are “passengers,” accumulated randomly with a background mutation rate (BMR) during cancer progression^2^. Moreover, it has been shown that the somatic mutation rates of cancer patients vary^3^. A patient with a high somatic mutation rate is referred as having a hypermutated phenotype. Environmental exposure^4^, DNA synthesis/repair dysfunction^3,5^, and regional mutation heterogeneity^3,6^ account for the mutational rate heterogeneity. For example, deleterious mutations in *POLE*, *POLD1*, and the MMR system defects may lead to a hypermutated phenotype^3,7^. Seven genes have been identified as essential components for MMR system, including *MLH1*, *MLH3*, *MSH2*, *MSH3*, *MSH6*, *PMS1*, and *PMS2*^8^.

Recently, immunotherapies targeting immune checkpoints, such as programmed cell death protein 1 (PD-1), along with its receptor (PD-L1), and cytotoxic T lymphocyte-associated antigen 4 (CTLA-4), have shown remarkable clinical benefits for various advanced cancers^9^. Nevertheless, only a fraction of patients are responsive to the treatment, making it critical to identify predictive biomarkers to distinguish responsive patients. PD-L1 expression level and microsatellite instability-high (MSI-H) have been used as predictive biomarkers for anti-PD-L1 therapy^10^. Microsatellite instability (MSI) is a phenotype of an accumulation of deletions/insertions in repetitive DNA tracts, called microsatellites. Similar to hypermutation, evidences have indicated that MSI results from a deficient MMR system^11^.

Hypermutation was first associated with the response to CTLA-4 blockade therapy in 2014^12^ and PD-1 blockade therapy in 2015^13^. Since then, tumor mutational burden (TMB), which is a measure of the abundance of somatic mutations, has become a new promising biomarker for both prognosis^14^ and immunotherapy^15^. Nevertheless, multiple challenges still hinder the adoption of TMB for clinical decision making. The major challenge is that even though the current well-accepted TMB measurement requires counting the non-synonymous somatic mutations in a paired tumor-normal sample using whole-exome sequencing (WES), current diagnostics based on sequencing technologies still rely heavily on targeted panel sequencing. Although studies have shown that panel-based TMB measurements were highly correlated with WES-based TMB^15–17^, inconsistencies between these two measurements have been observed^16–19^. One reason for this inconsistency is that targeted panel sequencing might overestimate TMB due to panel design biases, which aim for a high enrichment of mutation hot spots and driver mutations. In order to avoid overestimating TMB, various filtering strategies have been applied. For example, Foundation Medicine used COSMIC to filter out driver mutations and added synonymous mutations to reach an agreement with WES-based TMB^16^. These arbitrary filters are dependent on frequently updated databases, worsening the inconsistency, reproducibility and robustness of the calculation. Another non-negligible challenge is the relatively arbitrary selection of the TMB-high cutoff such as 10–20 per Mb or top 10–20% quantile^20^. Although these thresholds were enough to illustrate the predictive value of TMB as a biomarker, an adaptive TMB cutoff derived from relevant studies or clinical trials is still needed for robust performance.

**Figure 1:**
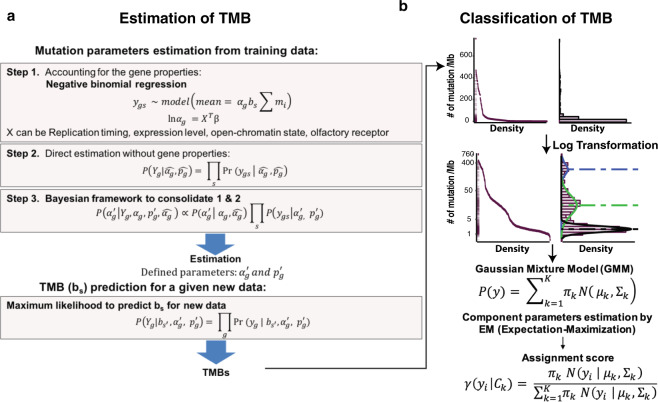
ecTMB workflow. **(a**) An explicit background mutation model for TMB prediction. (**b**) TMB subtype classification based on log-transformed TMB using GMM.

To improve the robustness of TMB measurement for targeted-panel sequencing and TMB subtype classification, here we propose a novel method called ecTMB (estimation and classification of TMB) (Fig. 1). While WES-based TMB is akin to the overall BMR due to the low incidence of driver mutation in whole exome, targeted sequencing panels require additional attention due to their size and targets. Our method uses a statistical model with a Bayesian framework for TMB prediction to systematically correct panel design biases. It was evaluated by assessing the agreement of the TMB prediction methods with the reference TMB from WES, by the TMB subtype classification accuracy with sliding TMB cutoffs, and by the classification assessment of subtypes from Gaussian Mixture Model (GMM) with WES data from The Cancer Genome Atlas (TCGA).

## Results

### Background mutation rate (BMR) modeling

BMR modeling is a major challenge for driver mutation detection and multiple methods using binomial^3^ and Poisson^21^ models have been developed for BMR; however, over-dispersion of mutation count has been observed in previous studies^22,23^. In our approach, we used a negative binomial model to capture the over-dispersion and assumed the occurrence of silent mutations follows the BMR in the absence of selection pressures. Studies have also shown that the BMRs vary as much as ~1000 fold, among cancer types, among patients for a given cancer type, and across different genomic regions. To incorporate all known cancer mutational heterogeneity factors (e.g. tri-nucleotide context^5^, sample-specific BMR, gene expression level^3^, and replication timing^24^), the Generalized Linear Model (GLM) was used to estimate the general impact of these factors by pooling genes together (Fig. 1a).

In order to evaluate our model, we divided samples corresponding to each cancer type into training and test sets with a 70%:30% split. GLM model parameters were determined from the training set, followed by the calculation of the number of background mutations of each gene for each sample (see Methods). Since synonymous mutations were accumulated with a BMR, the comparison of predicted number of synonymous mutations against observed was used to assess the performance of the model. We found that GLM could not explain all the variations in the observed number of synonymous mutations. For example, two suspicious false-positive driver genes^3^, membrane-associated mucin (MUC16) and titin (TTN), had much lower predicted number of synonymous mutations than actually observed in both the training and test sets (Supplementary Fig. 1). Therefore, there were likely unknown sequencing or biological factors influencing BMR as well.

To handle unknown factors, we separately modeled each gene as an independent negative binomial process. The final adjusted gene-specific BMRs were then generated through a Bayesian framework to consolidate the estimators from the previous two steps (Fig. 1a) (details in Methods). Compared with the prediction of synonymous mutations from GLM, the final model improved the R-squared from ~0.5 to ~0.9 in the training set and from ~0.3 to ~0.6 in the test set. It also reduced the mean absolute error (MAE) and the root mean square error (RMSE). The mutation predictions for MUC16 and TTN then became much closer to the observed values (Supplementary Fig. 1). In addition, the observed number of non-synonymous mutations in well-known cancer-specific driver genes, such as TP53, KRAS, and PIK3CA, were much higher than the predicted background ones due to positive selection (Supplementary Fig. 1). Our results showed that the three-step approach gained an improved performance overall.

Because other BMR modeling methods developed for driver gene detection^3,21,25,26^ only reported statistical p-values for driver genes instead of their predicted background mutation, we generated driver gene statistics (described in Supplementary Method) to compare ecTMB against other methods and used the enrichment of known cancer drivers from Cancer Gene Census (CGC)^27^ as a benchmark. The comparison demonstrated that our method performed the best (Supplementary Fig. 2).

### TMB prediction

In our BMR model, the trinucleotide BMR and gene-specific BMR can be determined from a training cohort. With the assumption that sample-specific BMR is equivalent to TMB, a new sample’s TMB could be calculated as the number of non-synonymous mutations per Mb, which is equivalent to TMB. With determined gene-specific BMRs from training set as described above, sample-specific BMR for a new sample could be estimated by Maximum Likelihood Estimation (MLE) through modeling each gene as an independent negative binomial process (Fig. 1a; see Methods).

Using our test sets, we evaluated the prediction performance of ecTMB by using all mutations, non-synonymous, and synonymous mutations from WES. ecTMB was compared against the standard TMB measurement (WES-based TMB), calculated by the number of non-synonymous mutations divided by the sequenced genomic region size. Even correlation coefficient (R) is widely used to assess the agreement of TMB measurements among assays, a high correlation does not mean two methods agree because R only measures the strength of a relation between two variables but not the exact agreement between them^28^. In order to comprehensively assess the agreement, we not only used Spearman correlation coefficient (R), but also measured R^2^, MAE, RMSE, slope of regression line, and standard error of regression (sigma), as well as performed Bland-Altman analyses. We found that the TMB predictions by ecTMB were highly concordant with standard TMB calculations, as exemplified in lung cancer (R = 0.99 and MAE = 0.31; see Supplementary Fig. 3).

ecTMB can use synonymous mutations for TMB prediction since synonymous mutations follow the background mutation accumulation. It is also able to incorporate non-synonymous mutations as most of which follow the BMR as well. We further assessed the impact of including non-synonymous mutations from different proportions of genes. Genes were ranked based on the ratio of observed vs expected number of mutations for each cancer type in the training set, with top ranked genes having higher likelihood to be driver genes. Then, non-synonymous mutations from the genes with the lowest ratios (bottom 0%, 20%, 40%, 60%, 80%, 95%, 99%, 99.9%, and 100%) were added to the prediction. While predictions with only synonymous mutations already achieved a great concordance with R > 0.975, the addition of non-synonymous mutations further improved the concordance, where R > 0.999 (Supplementary Fig. 3).

We further conducted *in silico* assessments of panel-based TMB prediction by three methods: (1) counting method; (2) counting after filtering COSMIC variants to remove known driver mutations; and (3) ecTMB. Six cancer panels were used, including Agilent ClearSeq, Illumina TruSight Tumor 170 (TST170), the 341-gene Integrated Mutation Profiling of Actionable Cancer Targets (MSK-IMPACT_341 gene)^29^, the 468-gene MSK-IMPACT, FoundationOne CDx, and Thermo Fisher Oncomine Tumor Mutation Load Assay (Oncomine-TML). The size of exonic region on the panels ranges from 400 kb to ~1.1 Mb (Supplementary Table 2). To evaluate panel TMB prediction, we only used cancer types with at least 10 samples with TMB > 10 in the test set (Supplementary Table 1), which included bladder urothelial carcinoma (BLCA), skin cutaneous melanoma (SKCM), lung adenocarcinoma (LUAD), lung squamous cell carcinoma (LUSC), colon adenocarcinoma (COAD), rectal adenocarcinoma (READ), stomach adenocarcinoma (STAD), uterine corpus endometrial carcinoma (UCEC), and head and neck squamous cell carcinoma (HNSC). We conducted the evaluations using both aggregated samples from all 9 cancer types and each cancer separately.

**Figure 2:**
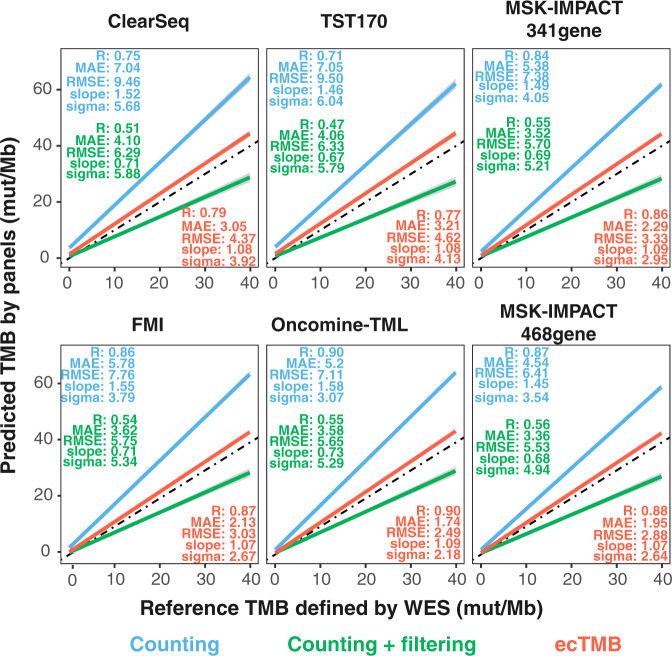
Evaluation of the panel-based TMB prediction performance. Plots show linear regression line of predicted panel-based TMBs against WES-based standard TMB for each panel using aggregated samples from 9 cancer types (in range of 0–40 mut/Mb WES-based TMB). Three methods were used for panel-based TMB predictions, including counting method (in cyan), counting with COSMIC filtering (in green) and ecTMB (in red). Their performance measurements (Spearman correlation coefficient, MAE, RMSE, slope and sigma) are plotted for each method in each plot.

**Figure 3:**
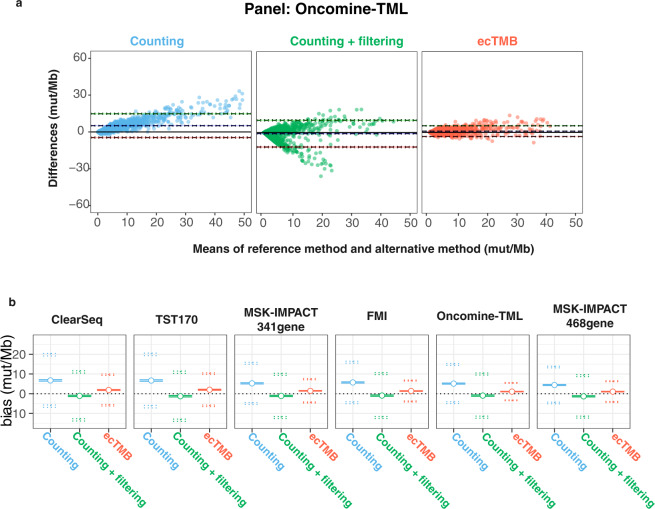
Bland Altman analysis of the panel-based TMB prediction against WES-based TMB. (**a**) Example Bland Altman plots for Oncomine TML panel show the means of WES-based TMB (reference method) and panel TMB prediction (alternative method) and differences of two methods. Three methods were used for panel-based TMB predictions, including counting method (in cyan), counting with COSMIC filtering (in green) and ecTMB (in red). The purple dashed line shows the estimated bias (mean difference). The green and pink dashed line show the upper limit of 95% agreement and the lower limit of 95% agreement, respectively. (**b**) A concise way to show Bland Altman analysis for all panels using aggregated samples from 9 cancer types (with WES-based TMB range 0–40 mut/Mb). The middle circle indicates the bias (mean difference) and two solid lines around it are 95% confidence interval for the bias. The two dotted line on the top are 95% confidence intervals for the upper limit of 95% agreement and ones on the bottom are 95% confidence intervals for the lower limit of 95% agreement.

Similar to other studies^15–17^, we detected a high correlation between WES-based standard TMB and panel-based TMB by simply counting mutations. The Spearman correlation coefficient (R) ranged from 0.71 to 0.9, and generally increased with the size of the panel (Fig. 2). The standard error of regression (sigma) decreased as panel size increased, indicating a narrower prediction interval for larger panel. Nevertheless, Bland-Altman analyses showed significant biases (>5) of counting method and a slope of ~1.5 for all six panels, indicating an over-estimation. Filtering out variants in COSMIC removed prediction biases but introduced more variation (Fig. 3), where a portion of samples got a higher reduction of mut/Mb than the rest (Supplementary Fig. 5). The non-uniform reduction of mut/Mb led to a higher standard error of regression (sigma) and a lower correlation score (Fig. 2).

In contrast, ecTMB predictions, using both synonymous and non-synonymous mutations, not only improved correlation coefficients with WES-based TMB, but also reduced MSE, RMSE, sigma, and biases while keeping the slope close to 1. As an example, for the predictions of the TST170 panel, ecTMB improved R from 0.71 to 0.77, reduced MAE from 7.05 to 3.21, and decreased sigma from 6.04 to 4.13 if compared with the counting method without filtering (Fig. 2). For the FMI panel, which is another large panel, ecTMB generated better TMB estimation (R = 0.87, MAE = 2.13, sigma = 2.67 and slope = 1.07) than the counting method (R = 0.86, MAE = 5.76, sigma = 3.79 and slope = 1.55) and counting with COSMIC filtering (R = 0.54, MAE = 3.62, sigma = 5.34 and slope 0.71). These performance metrics demonstrated that TMB prediction by ecTMB has a higher agreement with WES-based TMB. The evaluation for each cancer type can be found in supplementary figures (Supplementary Figs. 5 and 6).

### Classification performance by a range of TMB thresholds

**Figure 4:**
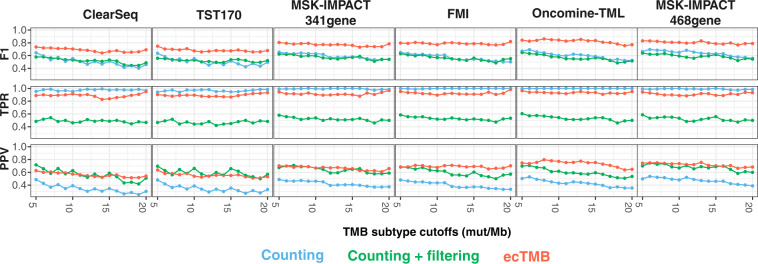
Classification performance by a range of TMB thresholds. WES-based TMB was used to define TMB subtypes by a series of thresholds as truth set. The same cutoff as WES-based TMB was used to determine panel-based TMB subtypes using panel-TMB prediction by three methods, including counting method (in cyan), counting with COSMIC filtering (in green) and ecTMB (in red). Classification accuracy from each panel-TMB was summarized in F1 score, TPR and PPV.

Although a wide range of cutoffs (5–20 mut/Mb) have been used to define samples with higher TMB in multiple clinical trials^20^, there is no definite threshold to classify TMB subtypes yet. Therefore, we evaluated the classification accuracy at multiple thresholds (ranging from 5 to 20) using aggregated samples from 9 cancer types with WES-TMB in 0–40 mut/Mb. Compared to subtypes defined by WES-based TMB, F1 score, true positive rate (TPR), and positive predictive value (PPV) were calculated for each threshold using the predicted panel-based TMB by the three methods aforementioned. A higher accuracy (F1 score) of classification was observed in larger panels for all the three methods. Counting method without filtering had the highest sensitivity (TPR) but consistently low precision (PPV). Counting method with COSMIC filtering showed low sensitivity but relatively good precision in small panels. ecTMB prediction had pretty good sensitivity and also high precision, leading to higher overall accuracy when comparing to the other 2 methods (Fig. 4). Within each cancer type, the same analyses were conducted, and ecTMB showed the best classification performance most of the time (Supplementary Fig. 7).

### Classification performance for subtype defined by GMM

**Figure 5:**
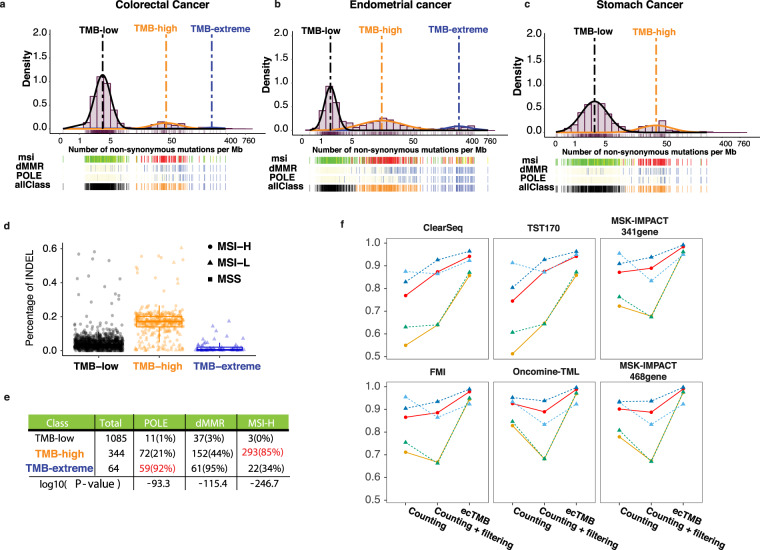
Three subtypes revealed by log-transformed TMB in colorectal, endometrial and stomach cancers. (**a–c**) Distribution plots of log-transformed TMB for (1) colorectal, (2) endometrial, (3) stomach cancers. Three subtypes were determined by Gaussian Mixture Model classification and labeled with black (TMB-low), orange (TMB-high) and blue (TMB-extreme) in bar named allClass. MSI status for each subject is shown with green (MSS) and red (MSI-H) in msi bar. Non-synonymous mutation existence (occurrence >1) in *POLE* or dMMR pathway genes, including *MLH1, MLH3, MSH2, MSH3, MSH6, PMS1, and PMS2* are shown in blue and wild type is shown in yellow. (**d**) Boxplot shows INDEL mutation percentages for three subtypes. (**e**) Tables shows total number of samples in each subtype, number of samples with at least one non-synonymous mutation in dMMR/*POLE* genes and number of samples with MSI-H status. The percentages of mutated *POLE*/MMR or MSI-H samples in each subtype are labeled in parentheses. Two-sided fisher exact tests were conducted to generate the P-value for each mutation profile among the subtypes. (**f**) The plots show the comparison of overall accuracy (red), overall kappa score (orange) and F1 score for each subtype (TMB-low in cyan, TMB-high in green and TMB-extreme in blue) for TMB subtype classification using TMB predicted by ecTMB or counting method.

While exploring the distribution of TMB, we discovered that the distribution of log-transformed WES-based TMB resembled a mixture of gaussians in colorectal, stomach, and endometrial cancers (Fig. 5a–c); however, we could not identify a similar pattern in other cancers (see Supplementary Method), indicating this may be unique to cancers with a high percentage of MSI-H cases. Because of the lack of the same pattern in those cancer types, we focused our analyses only on colorectal, stomach, and endometrial cancers. All three cancer types had at least two Gaussian clusters which consisted of TMB-low and TMB-high samples, respectively. In colorectal and endometrial cancers, there was a third Gaussian cluster, in which samples possessed extremely high TMB, namely TMB-extreme. We further classified each sample to these three subtypes by Gaussian Mixture Model (GMM) for investigation of their significance.

To gain insights into which mechanism may be responsible for the distinct TMB levels among the three subtypes, we examined the non-synonymous mutations in *POLE* and seven MMR genes, as well as their MSI status detected as described in earlier works^30–32^. We found that a large fraction (92%) of TMB-extreme samples possessed at least one non-synonymous mutation in *POLE* in aggregated colorectal, endometrial, and stomach cancer samples, among which we detected a high recurrence of 2 known *POLE* driver mutations (P286R and V411L)^7^ (Supplementary Fig. 10). Additionally, almost all of the TMB-high samples (85%) were MSI-High (MSI-H). The comparison of non-synonymous mutations in seven MMR genes between TMB-high samples against the rest revealed 2 highly recurred mutations: N674lfs*6 in MLH3 and K383Rfs*32 in MSH3, which have not been reported as driver mutation before (Supplementary Fig. 10). We also found that TMB-high samples generally had a significantly higher fraction (~17%) of INDEL mutations than what was observed in both TMB-low (~5%) and TMB-extreme (~1%) samples (Fig. 5d), consistent with the MMR defect phenotype. These distinct mutation profiles suggest that defective MMR system could be the likely cause for TMB-high whereas mutated *POLE* system for TMB-extreme. Additionally, the survival at different levels after considering other factors (hazard ratio (HR) for TMB-high = 0.79 with p-value = 0.067; hazard ratio (HR) for TMB-extreme = 0.41 with p-value = 0.033) (Supplementary Fig. 11). This observation was consistent with a previous study that revealed 8 major hypermutation clusters using trinucleotide mutation signature^7^, which included POLE-driven and MSI-driven hypermutation clusters. Our results illustrated that a simple log-transformation of TMB in colorectal, endometrial, and stomach cancers could also reveal POLE-driven and MSI-driven tumors.

With the discovery of biologically meaningful subtypes defined by the log-transformed TMB, we extended the capability of our method to classify TMB subtypes using GMM (Fig. 1). Using the subtypes determined by WES-based TMB as truth, we evaluated its classification accuracy in using ecTMB to predict panel-based TMB from the test set versus the counting method. When compared to the counting method, we found that classification using ecTMB improved not only the overall accuracy and kappa concordance score, but also the F1 score for each subtype classification (Fig. 5f).

## Discussion

We developed an explicit background mutation model for TMB prediction. To our knowledge, this is the first such approach for TMB prediction although BMR modeling has been widely used for driver mutation/gene/pathway detections. We have shown that our three-step BMR model predicted background mutations more accurately and highlighted well-known cancer-specific driver genes. Additionally, ecTMB has several advantages over existing counting method. First, ecTMB improves the consistency of TMB prediction among assays through systematic correction of panel design biases. Second, ecTMB takes synonymous mutations into account for TMB prediction, which improves the accuracy of panel-based TMB prediction. Last but not least, ecTMB predicts TMB by considering each gene as an independent negative binomial process, which provides a more robust prediction compared to predicting TMB based on a single counting value. Although there are other factors influencing the consistency of TMB among assays, such as sequencing depth and choice of somatic mutation caller, we have demonstrated that ecTMB can help to improve the stability of TMB measurement when those factors are fixed. It is possible that more factors can be added to our statistical framework to further improve the consistency of TMB measurements.

Although *POLE*-driven and MSI-driven hypermutation have been discovered by clustering mutations’ trinucleotide context before^7^, our work showed that a simple log-transformation of TMB can also reveal similar subtypes, i.e. TMB-low, TMB-high, and TMB-extreme. Our results indicated that TMB-high suffered from MMR system defects and TMB-extreme was likely caused by dysfunctional *POLE*, illustrating that MSI-H is one subtype of hypermutated tumor. The two novel driver mutations for MMR defects merit further research and clinical follow-up. Although multiple studies have observed a better prognosis in MSI-H patients, we showed that TMB-extreme caused by dysfunctional *POLE* showed even better overall survival outcomes compared to TMB-high (MSI-H). Similar to our result, studies have reported that mutations in *POLE* proofreading domain are associated with improved prognosis in several cancer types, including high-grade glioma^33^, lung adenocarcinoma^34^, endometrial cancer, and colorectal cancer^35^. This suggests that TMB-extreme might be another promising biomarker to predict patient prognosis or guide cancer treatment. The TMB-extreme subtype can also be potentially detected with an assay targeting only mutations in the *POLE*’s proofreading domain.

ecTMB is a robust and flexible statistical framework for TMB prediction and classification, shedding light on clinically relevant TMB subtypes. We believe that our method can help facilitate the adoption of TMB as biomarker in clinical diagnostics.

## Methods

### Datasets

Somatic mutations reported by MuTect2 (using the human genome reference build hg38) and clinical profiles of TCGA samples were downloaded from NCI Genomic Data Commons^36^ using TCGAbiolinks^37^. Formalin-fixed paraffin-embedded (FFPE) tissue samples were excluded from downstream analysis. The main cancer types included in our analyses were bladder urothelial carcinoma (BLCA), skin cutaneous melanoma (SKCM), lung adenocarcinoma (LUAD), lung squamous cell carcinoma (LUSC), colon adenocarcinoma (COAD), rectal adenocarcinoma (READ), stomach adenocarcinoma (STAD), uterine corpus endometrial carcinoma (UCEC), and head and neck squamous cell carcinoma (HNSC). Similar to earlier work, the pair of READ and COAD and the pair of LUAD and LUSC were combined for analysis due to their similarity^30,38^. The availability of MSI status of these cancer types provided us an opportunity to investigate the association between TMB and MSI status.

### Whole exome annotation

Ensembl build 81 (for the GRC38 human reference) was downloaded and processed to generate all possible mutations and their functional impacts for the genome. First, we changed every genomic base in coding regions to the other three possible nucleotides and used Variant Effect Predictor (VEP) to annotate their functional impacts. Each variant’s functional impact was chosen based on the following criteria: biotype > consequence > transcript length. Each variant’s tri-nucleotide contexts, including before and after the mutated base, and corresponding amino acid positions relative to protein length were reported.

### TMB prediction method

Our method adopted background mutation modeling and added a final step to predict TMB using maximum likelihood for a given new sample. A training cohort with whole-exome sequencing data can be used to estimate parameters of BMR model. The modeling steps of TMB prediction are detailed in the Supplementary Method.

Different proportions of non-synonymous mutations can be used for TMB prediction. In BLCA, SKCM, LUSC, and LUAD, which are known to harbor somatic mutations caused by environmental factors, the prediction accuracy increased as a higher proportion of non-synonymous mutations were included (Supplementary Fig. 4). However, the prediction accuracy in rest of the cancer types increased until 99.95% and then decreased when more non-synonymous mutations from genes with higher observed/expected ratio were added (Supplementary Fig. 4). Therefore, all non-synonymous mutations and synonymous mutations were used for TMB prediction in BLCA, SKCM, LUSC, and LUAD, whereas only non-synonymous mutations from the bottom 99.95% genes and all synonymous mutations were used for TMB prediction for the rest of the cancer types.

### TMB prediction evaluation

Bland-Altman analysis is a widely used method to assess the agreement between two different assays, providing a bias measurement (mean difference), the limits of agreement and 95% confidence intervals for these measurements. Bland-Altman analysis was done using the R package blandr.

Linear regression analysis was conducted using lm function in R.

Only samples with WES-TMB < = 40 were used for evaluation.

### TMB subtype classification using a series of threshold

For each threshold, WES-based TMB was used to define the true subtype of each sample. Then, we used the same threshold to determine the subtypes using predicted panel-based TMB. Classification accuracy measures were calculated by comparing the panel-predicted TMB subtype against the truth subtype defined by WES-based TMB.

Only samples with WES-TMB < = 40 were used for evaluation.

### TMB subtype classification using GMM

Log-transformed TMBs were modeled using Gaussian Mixture Model [1], in which components represent cancer subtypes. The Expectation-Maximization algorithm can be used to estimate each component’s parameters in the Gaussian mixture model with training data. The parameters for K^th^ component include weight (${\pi }^{k}$), mean (${\mu }^{k}$), and variance (${\varSigma }^{k}$). These parameters were used in assignment score calculation.

1 $$P(y)=\mathop{\sum }\limits_{k=1}^{K}\,{\pi }_{k}N({\mu }_{k},\,{\varSigma }_{k})$$

For a given new sample’s log-transformed TMB (${y}_{i}$), the assignment score for each component ($\,\gamma (b|{C}_{k})$) will be calculated as [2] using pre-defined parameters.

2 $$\gamma ({y}_{i}|{C}_{k})=\frac{{\pi }_{k}\,N({y}_{i}|{\mu }_{k},\,{\varSigma }_{k})}{{\sum }_{k=1}^{K}\,{\pi }_{k}\,N({y}_{i}|{\mu }_{k},\,{\varSigma }_{k})}$$

The new sample would be classified into the component which has the highest assignment score. Currently, weight = 1 was assigned to each component due to under-representation of TMB high samples.

### Cancer subtype classification and characterization

Within each cancer type (colorectal, endometrial, and stomach cancer), log-transformed TMBs, either defined by total number of mutations per Mb or number of non-synonymous mutation per Mb, were modeled using Gaussian Mixture Model as described above. Each sample was assigned to one of TMB-low, TMB-high and TMB-Extreme classes based on its assignment score. For each sample, INDEL incidence, estimated immune cell abundance and non-synonymous mutation existence (occurrence >1) in *POLE* and dMMR pathway genes including *MLH1, MLH3, MSH2, MSH3, MSH6, PMS1*, and *PMS2* were summarized. Mutations of *POLE* and MMR system genes were plotted using maftools^39^.

### TMB prediction for panels

In order to evaluate ecTMB prediction for panels, we performed *in silico* analyses for panels which we could get either target bed files or gene list from public domain. We downloaded panel coordinates bed files, including Illumina TruSight Tumor 170 from Illumina website, ClearSeq from Agilent website, Oncomine Tumor Mutation Load Assay from Thermo Fisher, and the 341-gene Integrated Mutation Profiling of Actionable Cancer Targets (MSK-IMPACT 341 gene) from publication^29^. The gene lists of FoundationOne CDx and the 468-gene MSK-IMPACT were download from the Foundation Medicine website (https://www.foundationmedicine.com/genomic-testing/foundation-one-cdx) and FDA (https://www.accessdata.fda.gov/cdrh_docs/reviews/den170058.pdf), respectively. Corresponding panel coordinates bed files were generated based on gene lists for FoundationOne CDx and MSK-IMPACT. Due to the lack of exact panel coordinates of FoundationOne CDx and MSK-IMPACT, the sizes of the panels converted from gene lists were different from the real commercial panels.

Mutations located in a given panel were selected to represent the mutations which can be detected by targeted sequencing for that panel. Within each cancer type, WES data of training set were used to determine background mutation model parameters. *In silico* performance evaluations were done using the test data. Both ecTMB and counting methods were applied to test data.

## Supplementary information

Supplementary Information.

## Data Availability

ecTMB is available at https://github.com/bioinform/ecTMB under the Creative Commons Attribution-NonCommercial-ShareAlike 4.0 International license.

## Data availability

Data for this study was generated by TCGA, downloaded from NCI Genomic Data Commons (https://gdc.cancer.gov/). Replication timing, expression level, and open-chromatin state of all protein-coding genes were extracted from Lawrence *et al*.^3^ (https://www.nature.com/nature/journal/v499/n7457/extref/nature12213-s2.xls). Illumina TruSight Tumor 170 panel coordinates bed file was downloaded from Illumina’s website (https://support.illumina.com/content/dam/illumina-support/documents/downloads/productfiles/trusight/trusight-tumor-170/tst170-dna-targets.zip) and the gene lists for FoundationOne CDx and MSK-IMPACT were downloaded from Foundation Medicine’s website (https://www.foundationmedicine.com/genomic-testing/foundation-one-cdx) and the FDA’s website (https://www.accessdata.fda.gov/cdrh_docs/reviews/den170058.pdf), respectively.
