# Supplementary material for: ecTMB: a robust method to estimate and classify tumor mutational burden
Source: Sci Rep. 2020 Mar 18;10:4983. doi: 10.1038/s41598-020-61575-1 (PMC7080796; doi:10.1038/s41598-020-61575-1)
Supplement: Supplementary file 1 — Supplementary Information. [file 41598_2020_61575_MOESM1_ESM.pdf]

ecTMB: a robust method to estimate and classify tumor mutational burden

Supplementary Information

Lijing Yao[1], Yao Fu[1], Marghoob Mohiyuddin[1], Hugo Y.K. Lam[1*]

[1]Roche Sequencing Solutions, Santa Clara, CA 95050 USA

Corresponding author: Hugo Y.K. Lam, Roche Sequencing Solutions, 2841 Scott Blvd, Santa Clara, CA 95050 United States; bioinformatics.red@roche.com

Supplementary Figures

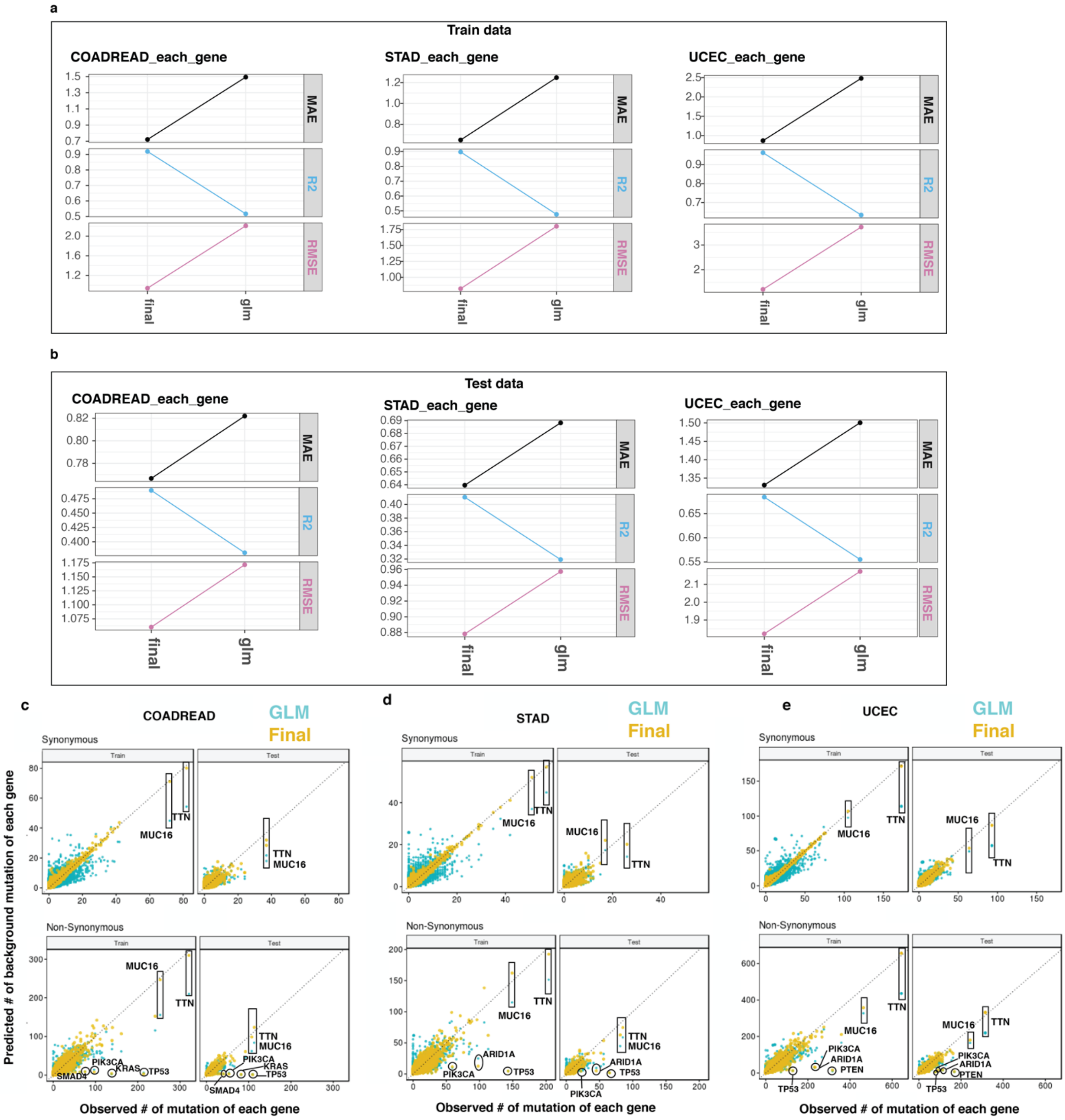

Supplementary Fig. 1 – Background mutation modeling comparison. (a-b) Plots show the comparisons of model accuracy between the GLM and the final (3-steps) approach in training sets (a) and in test sets (b). RMSE, MAE and R-squared were calculated between predicted number of synonymous mutations and observed value for each gene in aggregated samples. (c-e) Predicted number of background synonymous (top)/non-synonymous (bottom) mutation of each gene are plotted against observed ones in colorectal (c), stomach (d), and endometrial (e) cancers. The prediction made by GLM model is labeled in cyan and final (3-steps) approach in yellow. Several well-known driver genes are circled/labeled in the non-synonymous figures.

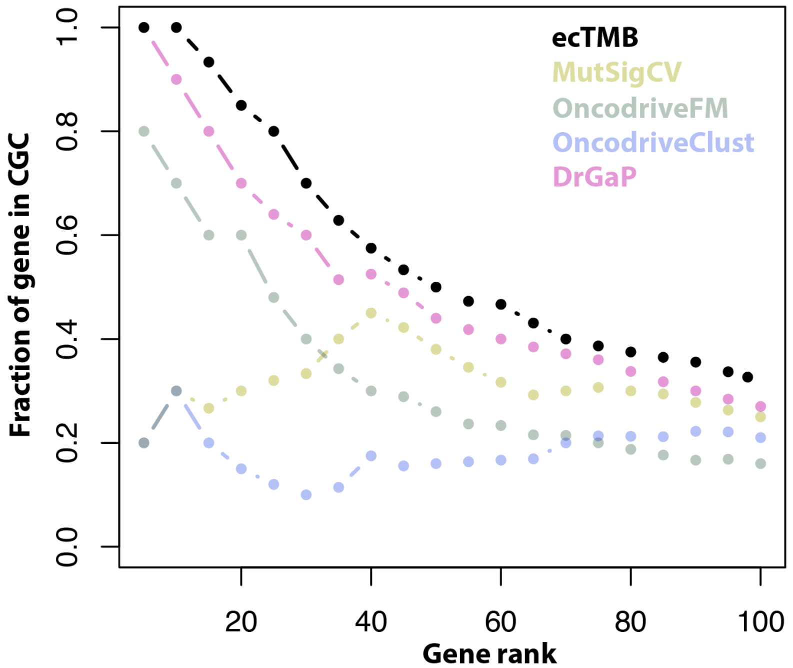

Supplementary Fig. 2 – Enrichment of detected drivers in Cancer Gene Census (BRCA).
Enrichment of genes (ranked by p-values) in Cancer Gene Census (CGC) from driver gene detection methods.

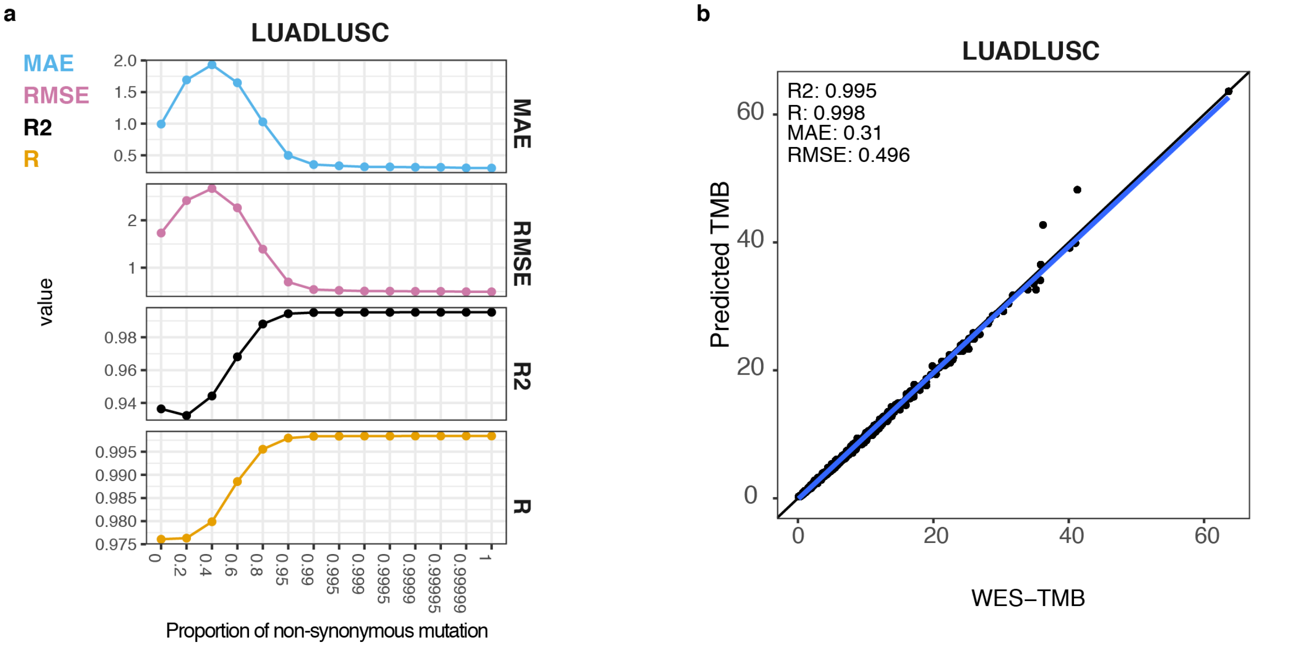

Supplementary Fig. 3 – ecTMB performance using WES data in Lung. (a) Plots show the comparisons of prediction accuracy when different proportions of non-synonymous mutation were used. RMSE, MAE and correlation coefficient were calculated between predicted TMB and standard WES-based TMB (mut/Mb). (b) Predicted TMB values are plotted against standard WES-based TMB. The linear regression lines were added. Standard WES-based TMB was calculated by counting the number of non-synonymous mutations and then divided by size of the exome (mut/Mb).

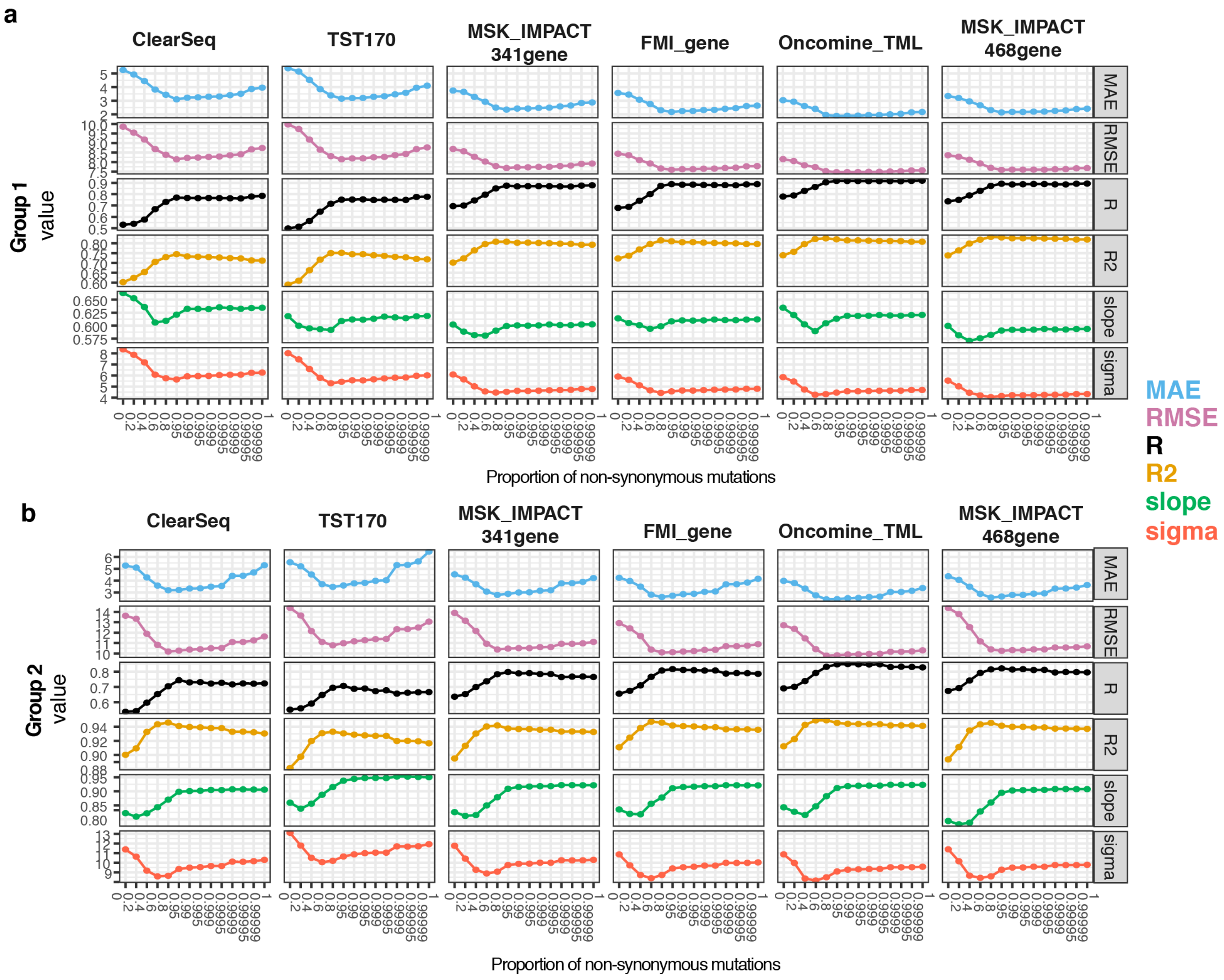

Supplementary Fig. 4 – Panel TMB prediction by ecTMB performance with different proportions of non-synonymous mutations. Plots show the comparisons of prediction accuracy when different proportions (0, 0.2, 0.4, 0.6, 0.8, 0.95,0.99, 0.995, 0.999, 0.9995, 0.9999, 0.99995, 0.999999, 1) of non-synonymous mutations were used for each panel. RMSE, MAE, correlation coefficient, sigma and slope were calculated between predicted panel-based TMB and standard WES-based TMB (mut/Mb). Group1 includes BLCA, SKCM, LUSC and LUAD and group2 includes COAD, READ, STAD, UCEC and HNSC.

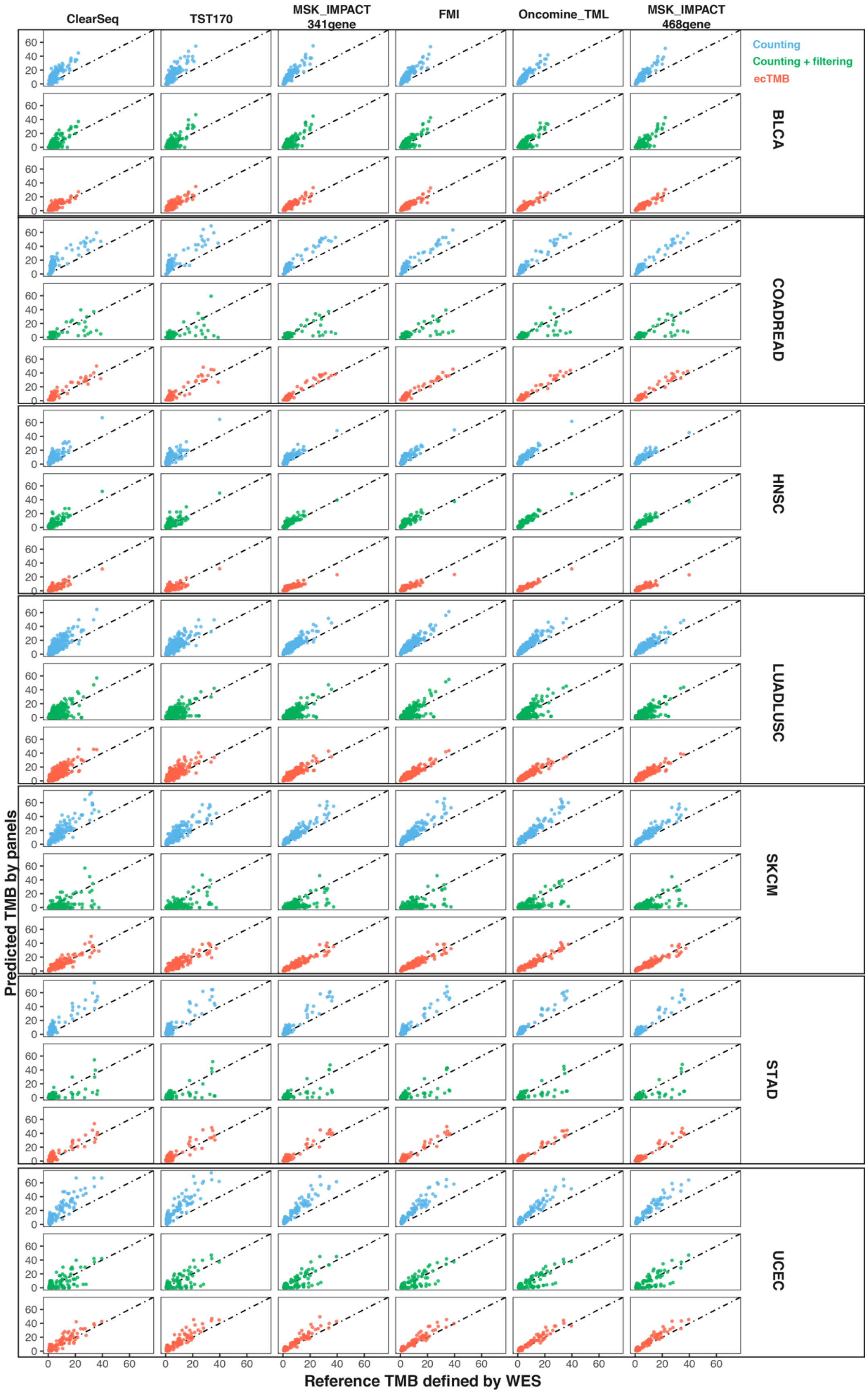

ClearSeq
TST170
MSK_IMPACT 341gene
FMI
Oncomine_TML
MSK_IMPACT 468gene
Counting
Counting + filtering
ecTMB
BLCA
COADREAD
HNSC
LUADLUSC
SKCM
STAD
UCEC
Predicted TMB by panels
Reference TMB defined by WES

Supplementary Fig. 5 – Scatter plot of predicted panel-based TMB and WES-based TMB for each cancer. (a) Scatter plots show WES-based standard TMB (mut/Mb) plotted against predicted panel-based TMBs (mut/Mb) for each cancer type and each panel. Three methods were used for panel-based TMB predictions, including counting method (in cyan), counting method with COSMIC filtering (in green) and ecTMB (in red).

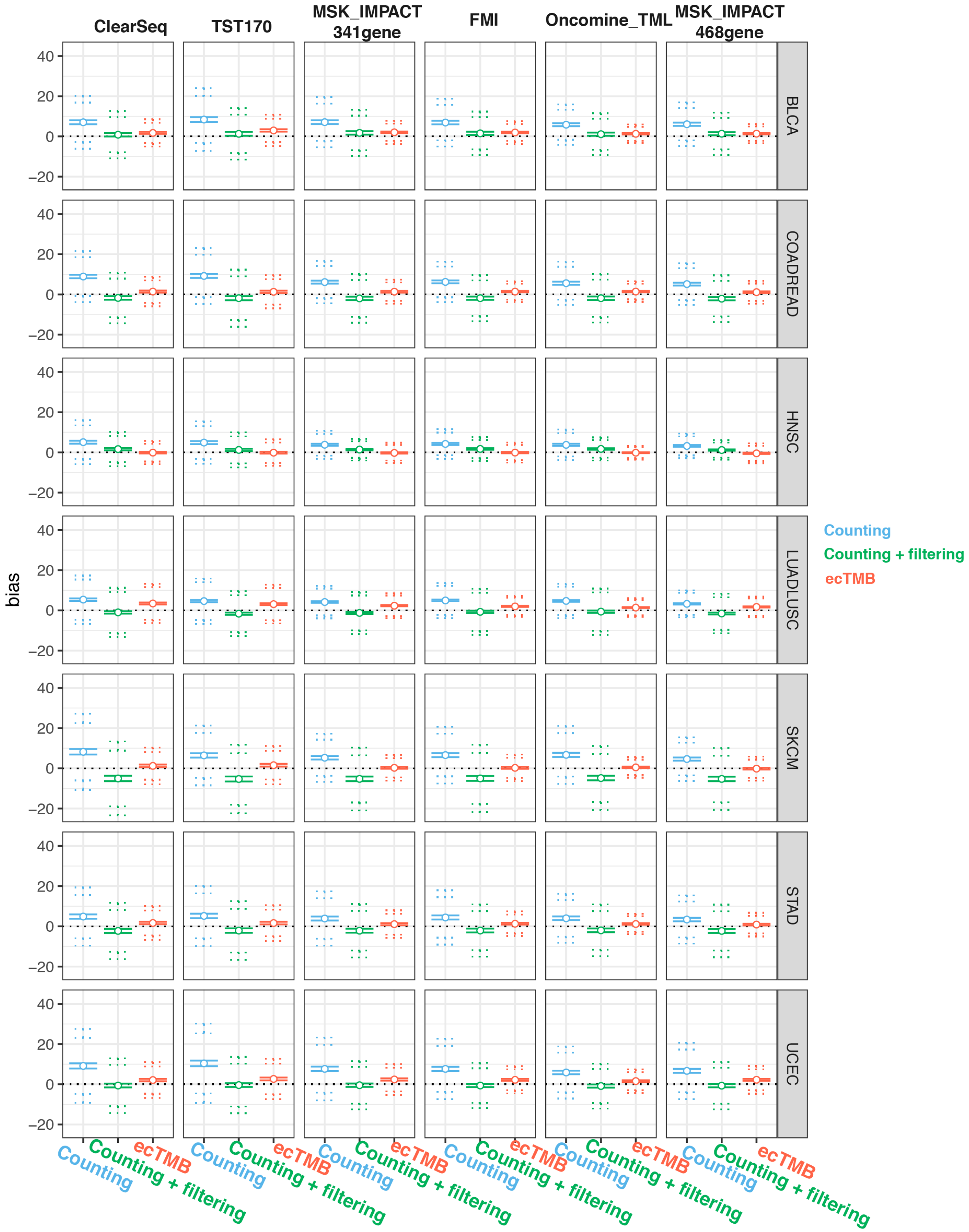

Supplementary Fig. 6 – Bland Altman analysis for each cancer type. Plots use a concise way to show Bland Altman analyses for all panels using aggregated samples from 9 cancer types. The middle circle indicates the bias (mean difference) and two solid lines around it are 95% confidence interval for the bias. The two dotted line on the top are 95% confidence intervals for the upper limit of 95% agreement and ones on the bottom are 95% confidence intervals for the lower limit of 95% agreement.

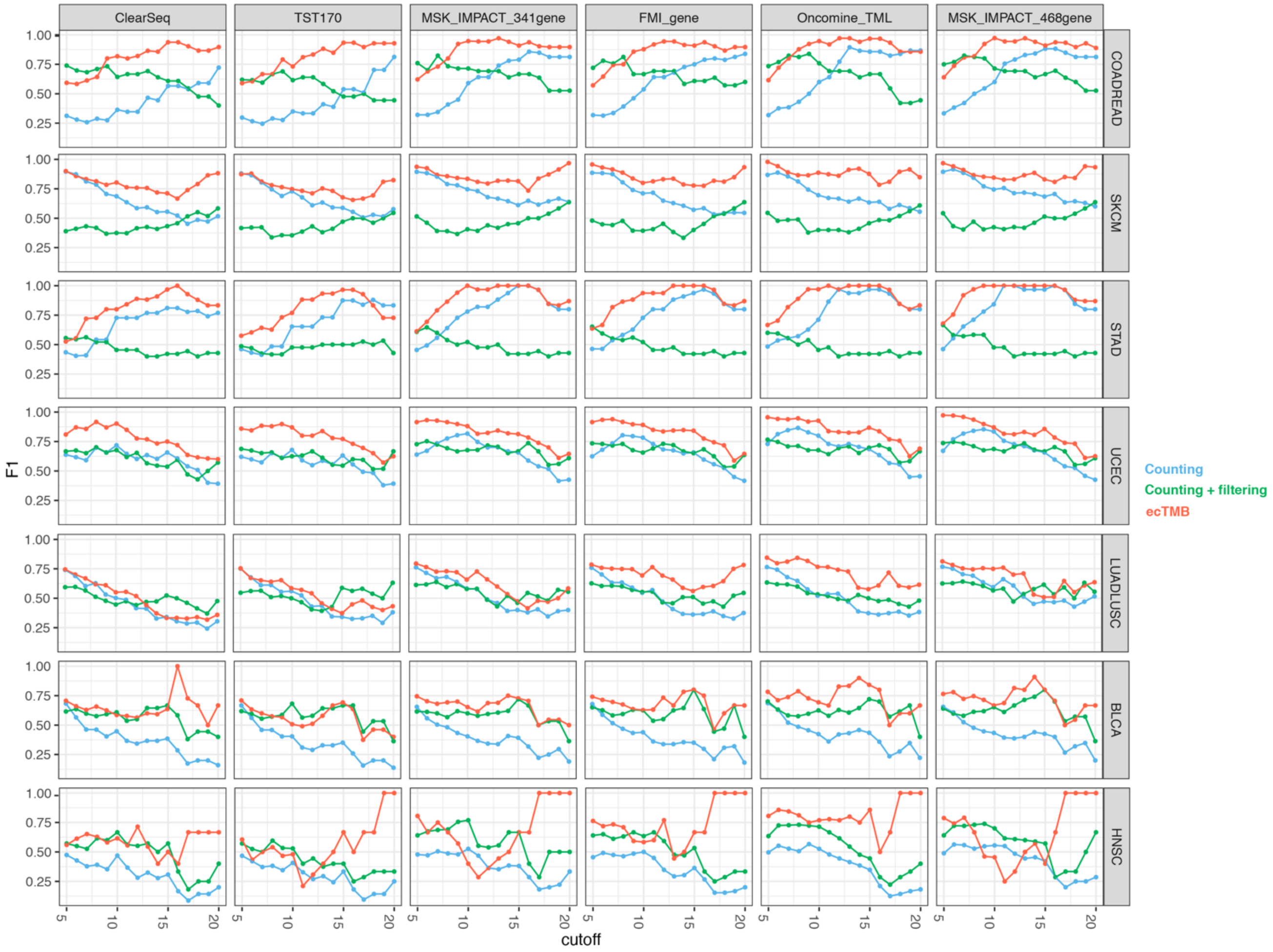

Supplementary Fig. 7 – Classification performance by a range of TMB threshold for each cancer type. WES-based TMB was used to define TMB subtypes by a series of threshold as truth set. The same cutoff as WES-based TMB was used to determine panel-based TMB subtype using panel-TMB prediction by three methods, including counting method (in cyan), counting with COSMIC filtering (in green) and ecTMB (in red). Classification accuracy from each panel-TMB were summarized in F1 score.

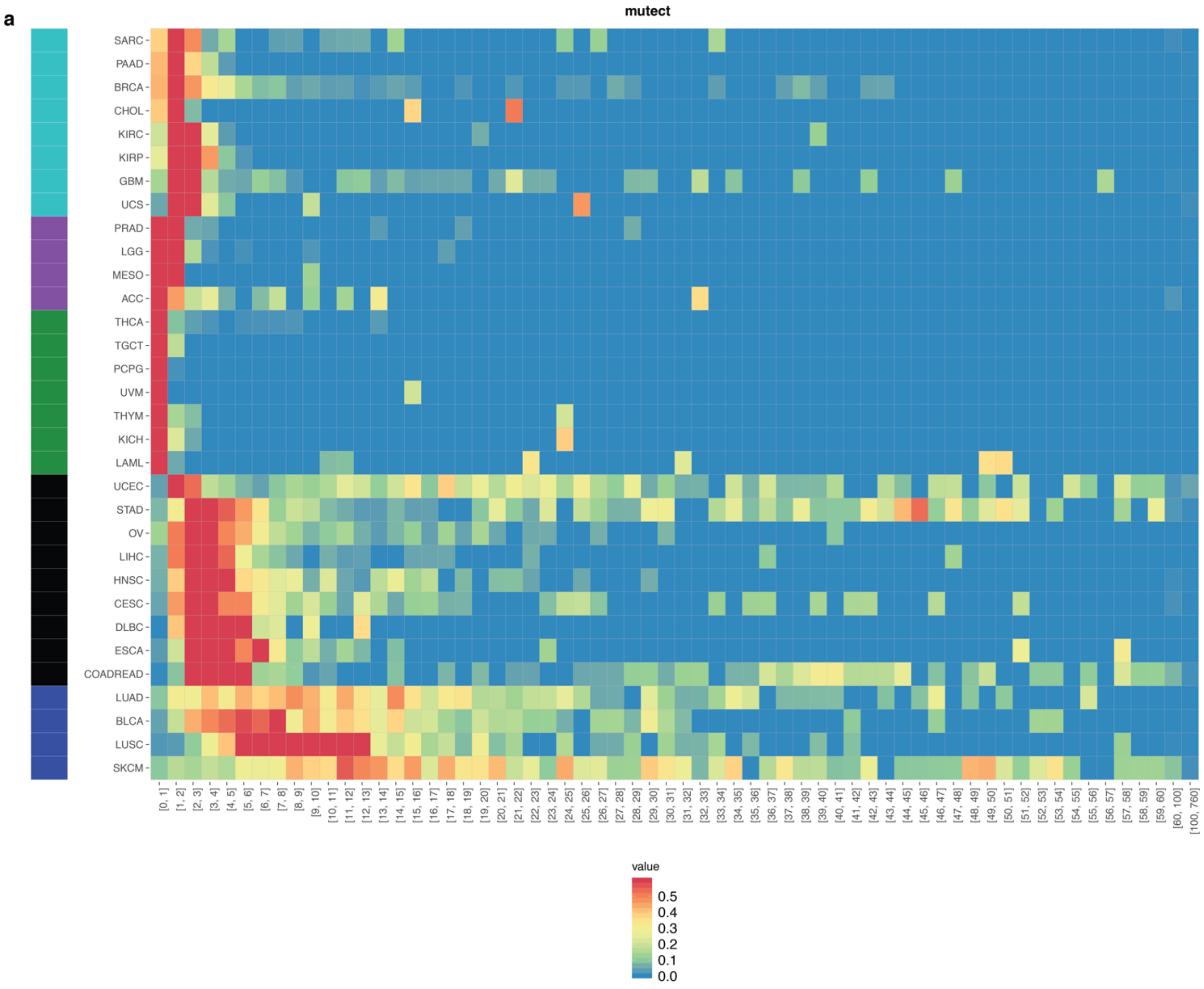

Supplementary Fig. 8 – Cancer groups based on distribution of log-transformed TMB. (a) Heatmap of distribution of log-transformed TMB. K-means clustering method was used to generate 5 clusters which is shown on the left side.

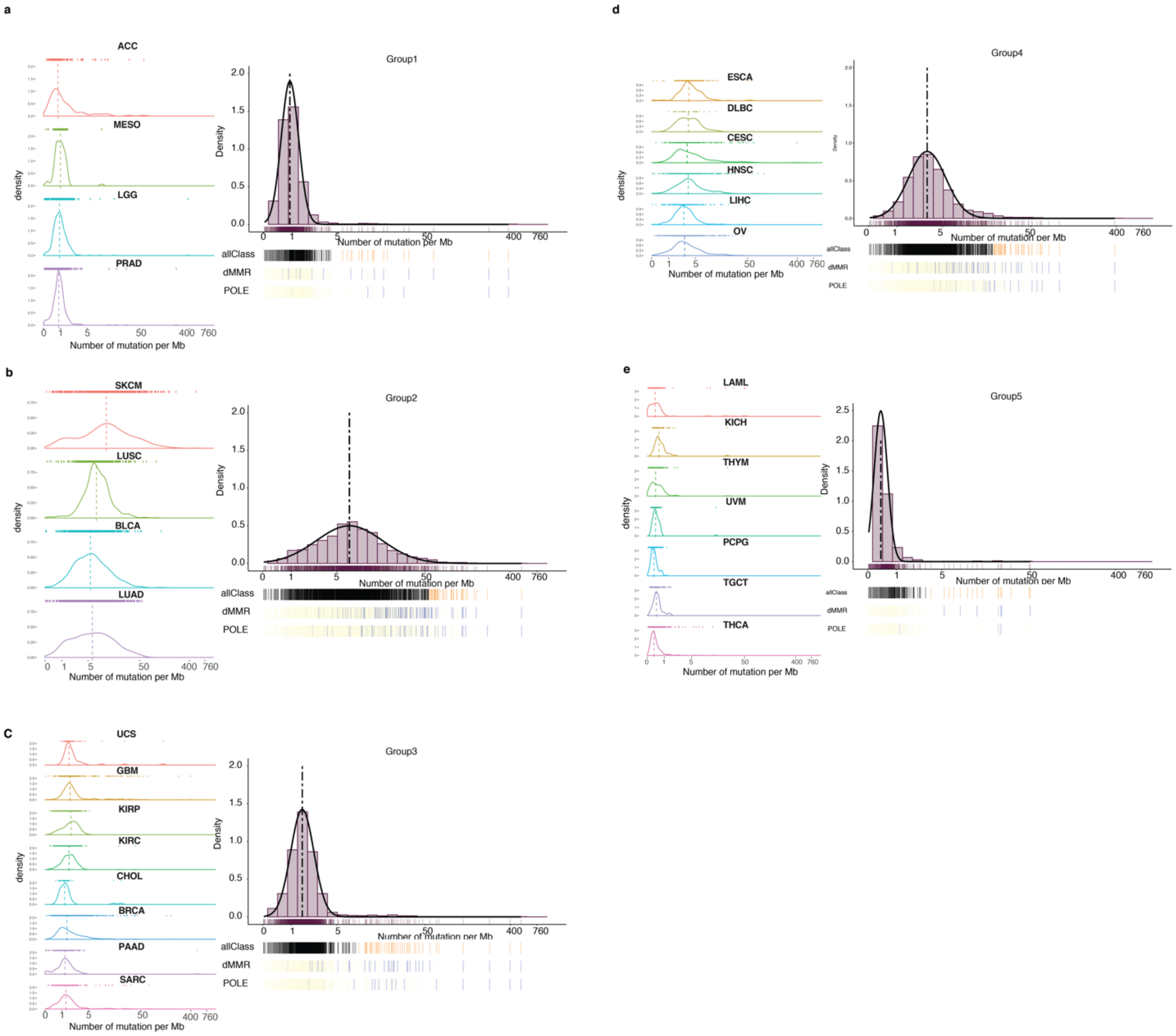

Supplementary Fig. 9 – Distribution for each cancer group. (a-e) Distribution of log-transformed TMB for each cancer: group 1 (a), group 2 (b), group 3 (c), group 4 (d) and group 5 (e). The distribution of log-transformed TMB for each individual cancer in each group is shown on the left.

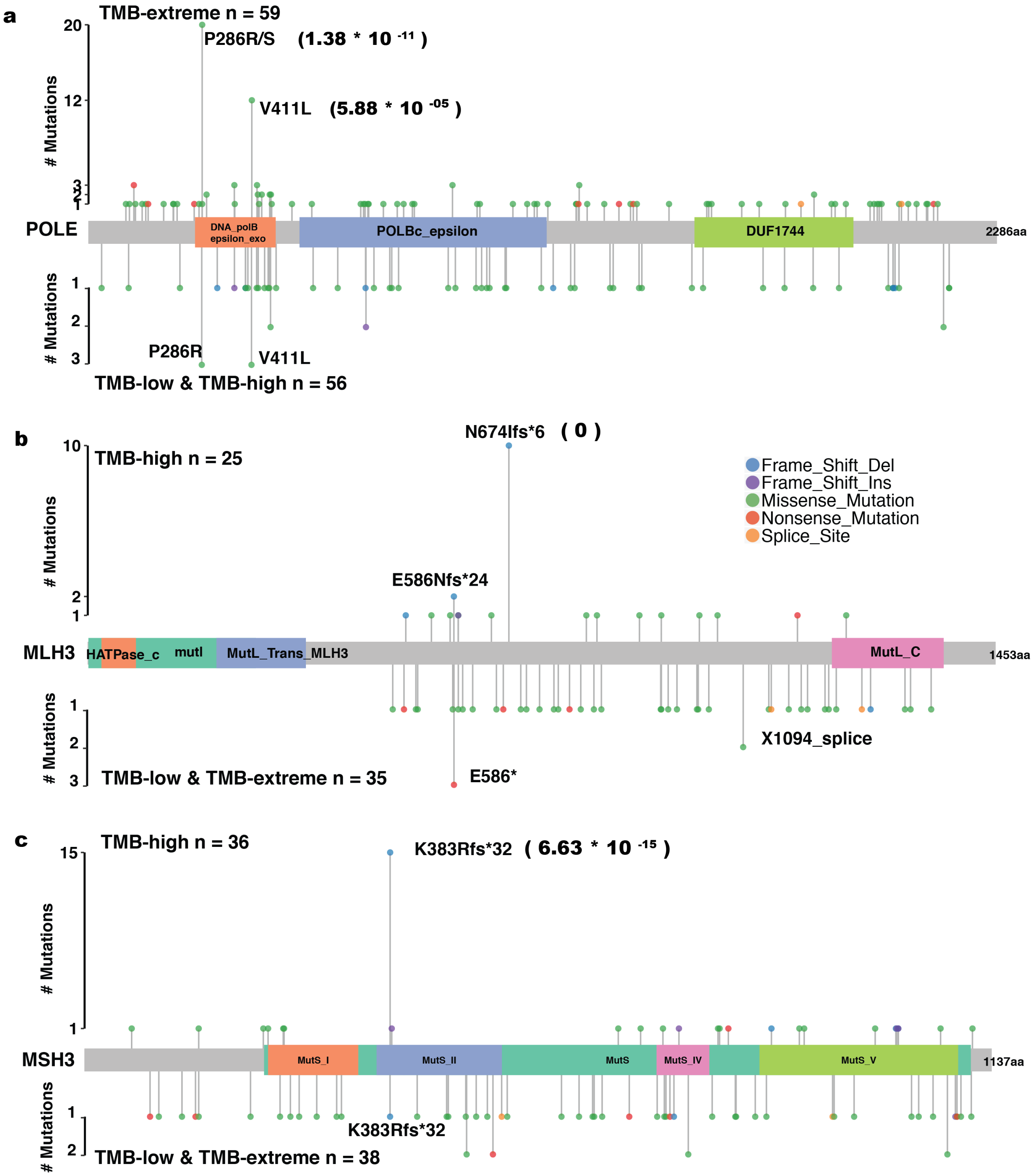

Supplementary Fig. 10 – Known and novel POLE, MLH3 and MSH3 driver mutations. (a) Landscape of driver mutations in POLE detected in TMB-extreme group (top) compared with aggregated TMB-high and TMB-low group (bottom). Enrichment p-values using two-sided binomial test are shown in parentheses. (b-c) Landscape of driver mutations in MLH3 and MSH3 detected in TMB-high group (top) compared with aggregated TMB-extreme and TMB-low group (bottom). Enrichment p-values using binomial test is shown in parentheses.

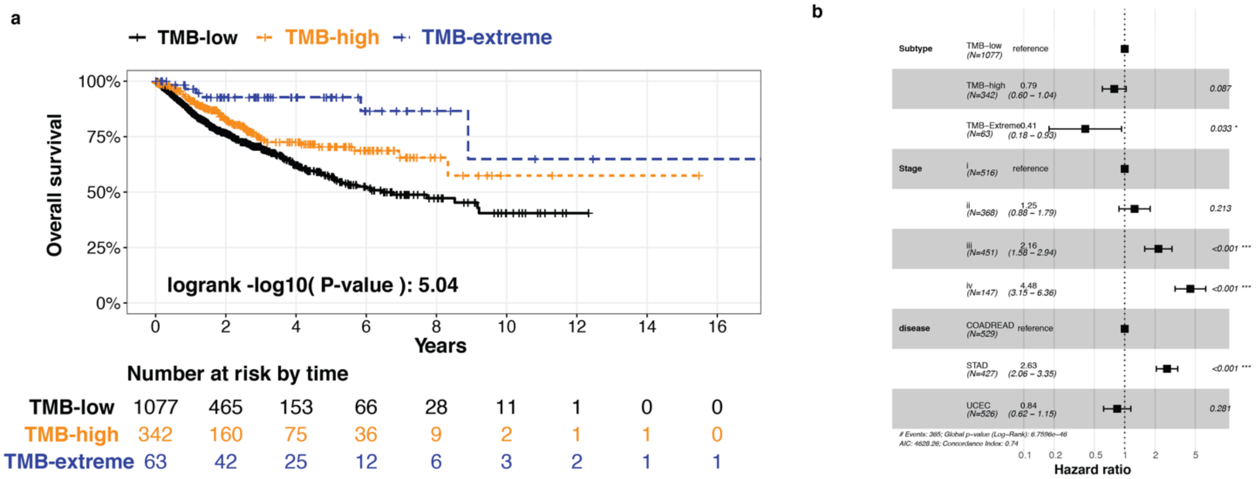

Supplementary Fig. 11 – Survival outcome association with three subtypes. (a) Kaplan-Meier survival curves stratified by TMB subtypes using aggregated colorectal, endometrial and stomach patients. (b) Proportional hazard ratio analysis by Cox proportional-hazards model.

Supplementary Tables

Supplementary Table 1: Number of test samples with TMB > 10

Cancer	TMB <= 10	TMB > 10
SKCM	100	56
LUADLUSC	278	56
UCEC	121	55
COADREAD	144	33
STAD	124	21
BLCA	119	18
HNSC	154	15
CESC	88	8
GBM	130	8
OV	138	7
BRCA	322	6
SARC	79	2
ESCA	59	2
LAML	48	2
LIHC	119	1
PAAD	59	1
CHOL	15	1
KIRC	112	1
UCS	19	1
ACC	31	1
THCA	164	1
PRAD	165	0
LGG	169	0
MESO	27	0
TGCT	43	0
PCPG	60	0
UVM	27	0
THYM	41	0
KICH	22	0

Supplementary Table 2: Size of exonic region on each panel

Panel	Size (bp)
**ClearSeq**	403504
**TST170**	403799
**MSK_IMPACT_341gene**	911535
**FMI_gene**	912660
**Oncomine_TML**	1169757
**MSK_IMPACT_468gene**	1189026

Supplementary Method

Detailed description of the TMB prediction method

1. Mutation rate for each sample (b_s)

The mutation rate for each sample (b_s) can be determined by the total number of mutations of the sample divided by the size of the evaluated genomic regions in Mb (Megabase) unit. If only non-synonymous mutations are used, b_s is equivalent to current standard TMB calculation. We use the number of non-synonymous mutation for the following TMB prediction and classification.

2. Tri-nucleotide context-specific mutation rates

Tri-nucleotide context-specific mutation rates are estimated for the training cohort. We consider the 96 possible tri-nucleotide contexts (from the 6 possible types of single base substitutions: A/T->G/C, T/A->G/C, A/T->C/G, T/A->C/G, A/T->T/A, G/C->C/G) and possible nucleotides around it, plus indels. Mutations are classified as synonymous or non-synonymous based on whether they have caused a change in the amino acid sequence of the translated protein. We assumed that synonymous mutations occur according to the BMR.

For each tri-nucleotide mutation context i, we calculate the number of synonymous $n_{i(synonymous)}$ and non-synonymous $n_{i(nonsynonymous)}$ mutations observed across all tumor samples and the number of possible synonymous $N_{i(synonymous)}$ and non-synonymous $N_{i(nonsynonymous)}$ variants in the exome. For non-synonymous mutations, only genes that are less likely to be drivers, are taken into consideration to avoid skewing the background non-synonymous mutation rate; i.e., the bottom 60% of genes ranked by the number of mutated samples in decreasing order. The potential bias introduced by using a subset of genes for non-synonymous mutations is corrected by the factor r, which is estimated using the method of moment[1], calculated as the mean of $\frac{n_{i(nonsynonymous)} / N_{i(nonsynonymous)}}{n_{i(synonymous)} / N_{i(synonymous)}}$ across all mutation contexts. For mutation context i, the mutation rate m_i is calculated as shown in Equation 1. When calculating indel

mutation rate m_{indel}, we assume that all protein-coding positions can have indels, and that all indels are considered as non-synonymous.

$$m_i = \frac{n_i(synonymous) + \ n_i(nonsynonymous)}{N_i(synonymous) + r \ \times \ N_i(nonsynonymous)} \qquad [1]$$

3. Gene-specific mutation rate α_g

3.1 Regression model across genes

We assume that the occurrence of synonymous mutations represents the background mutation rate, and we can model the number of synonymous mutations per gene using the negative binomial.

We consider multiple factors that could influence the underlying mutation rate to model the synonymous mutation counts. Firstly, the number of possible synonymous mutations is controlled by the gene's coding sequence (e.g. codons and length). Specifically, for gene g, we get all possible bases that could mutate to synonymous mutations and sum up their context-specific mutation rate as $E_{g(synonymous)} = \sum_{synonymous\ base} m_i$. Secondly, because different individuals are expected to have different background mutation rates, we use the aforementioned sample specific factor b_s to represent the total mutation burden of sample s. Thirdly, α_g is gene-specific mutation rate, influenced by several additional known factors that can influence the underlying mutation rate for a given gene, including replication timing (R), expression level (X), open-chromatin state (C), and whether gene is an olfactory receptor (O). The effect of these factors is estimated using negative binomial regressions as described below.

We model the synonymous mutation count y_{gs} of gene g and sample s with negative binomial regression [2], assuming common dispersion p across genes.

$$y_{gs} \sim NB\left(mean = \ \alpha_g b_s E_{g(non-synoymous)}, \ dispersion = \ p\right) \qquad [2]$$

Where

$$ln\alpha_g \ = \beta_0 + \ \beta_1 R_g + \beta_2 X_g + \beta_3 C_g + \beta_4 O_g$$

$\beta = \{\beta_0, \beta_1, \beta_2, \beta_3, \beta_4\}$ is estimated by performing regression with all genes.

3.2 Capture unknown factor impact through Maximum Likelihood Method

In [2] we assume mutation rate factors only depend on proposed regressors, but unknown mechanisms or biological factors can also impact mutation rate. Therefore, we model each gene as an independent zero-inflated Poisson process and use the Maximum Likelihood estimation (MLE) to estimate gene-specific dispersion parameter $\widehat{p_g}$ and gene-specific mutation rate $\widehat{\alpha_g}$ by maximizing [3].

$$\left(Y_g | \widehat{\alpha_g}, \widehat{p_g}\right) = \prod_s \frac{\Gamma\left(y_{gs} + \widehat{p_g}^{-1}\right)}{\Gamma\left(\widehat{p_g}^{-1}\right)\Gamma\left(y_{gs}+1\right)} \left(\frac{1}{1+\widehat{\alpha_g} b_s E_{g(non-synonymous)} \widehat{p_g}^{-1}}\right)^{\widehat{p_g}^{-1}} \left(\frac{\widehat{\alpha_g} b_s E_{g(non-synonymous)}}{\widehat{p_g}^{-1} + \widehat{\alpha_g} b_s E_{g(non-synonymous)}}\right)^{y_{gs}} \quad [3]$$

For each gene, $Y_g = \{y_{g1}, y_{g2}, \ldots, y_{gs}\}$ are the silent mutation counts in different samples, the initial value of $\widehat{\alpha_g}$ is given by $\widehat{\alpha_g} = \frac{1}{s}\sum_1^s \frac{y_{gs}}{b_s E_{g(synonymous)}}$, and the influencing factors (R, X, C, O) are not applicable.

3.3 Optimization of gene-specific mutation rate factors

Because we obtain α_g by pooling all genes together, it captures the common trend of the influencing factors (R, X, C, O) on background mutation rate. On the contrary, $\widehat{\alpha_g}$ is a gene-specific parameter from the observed data independent of the influencing factors. $\widehat{\alpha_g}$ and α_g are not always the same, which could be caused by technical noise (e.g. errors in mutation calling algorithms) or reflect real biological mechanisms (e.g. factors influencing the background mutation rate that are not included in our regression model). Due to the low number of somatic mutations in each gene, $\widehat{\alpha_g}$ is very vulnerable to statistical noise. Thus, we try to find the optimized α'_g by incorporating both parameters from negative binomial regression and those directly from

gene-specific estimation. The posterior probability of α'_g is proportional to the likelihood times prior with σ estimated as [7]. The prior probability is chosen to constrain α'_g to be centered at α_g. We maximize [4] to obtain the proper α'_g for each gene.

$$P(Y_g, \alpha_g, p'_g, \widehat{\alpha_g}) \propto P(\alpha'_g | \alpha_g, \widehat{\alpha_g}) \prod_s P(y_{gs} | \alpha'_{g,} \ p'_g) \quad [4]$$

$$y_{gs} \sim NB(\alpha'_g \, b_s E_{g(synonymous)} \, , p'_g) \quad [5]$$

$$P(\alpha'_g | \alpha_g, \widehat{\alpha_g}) = \frac{1}{\sigma\sqrt{2\pi}} e^{\frac{-(ln\, \alpha'_g - ln\, \alpha_g)^2}{2\sigma^2}} \quad [6]$$

$$\sigma = \sqrt{\frac{1}{N} \sum_g (ln\, \alpha_g - ln\, \widehat{\alpha_g})^2} \quad [7]$$

Then the 'gene-specific estimation' and 'optimization of gene mean' steps are repeated by replacing $\widehat{\alpha_g}$ with α'_g to re-estimate dispersions until convergence is achieved. The estimated α'_g and p'_g are used in the following steps.

4. TMB prediction

Using pre-defined α'_g and p_g, we model each gene as an independent negative binomial process for a given new sample s'. Then, we use Maximum Likelihood estimation (MLE) to estimate $b_{s'}$by maximizing [8]. In this step, $Y_g = \{y_1, y_2, \dots, y_g\}$ captures the synonymous mutation counts, and additionally, non-synonymous mutation counts in sample s'. When WES is used, Y_g includes all gene in the genome. When particular panel is used, $Y_g$includes the genes covered by the panel.

$$(Y_g | b_{s'}, \alpha'_g, p'_g) = \prod_g \frac{\Gamma(y_g + p'^{-1}_g)}{\Gamma(p'^{-1}_g)\Gamma(y_g+1)} \left(\frac{1}{1+\alpha'_g b_s E_{g(non-synonymous)} p'^{-1}_g}\right)^{p'^{-1}_g} \left(\frac{\alpha'_g b_s E_{g(non-synonymous)}}{p'^{-1}_g + \alpha'_g b_s E_{g(non-synonymous)}}\right)^{y_g} \quad [8]$$

Poisson and zero-inflated Poisson regressions can also be used for background mutation modeling, which is also implemented in the package. We used negative binomial for all analyses presented in this study.

Cluster cancer types based on TMB distribution

WES mutation data for 29 cancer types other than colorectal, endometrial, and stomach cancers were downloaded from GDC. However, many cancer types, such as adrenocortical carcinoma (ACC), do not have a significant number of hypermutated samples. In order to have a large population of hypermutated samples, we considered aggregating cancer types together. However, we discovered that mutation spectra among cancer types were different as well, indicating a different threshold for hypermutated population for each cancer. For example, the median mutation rate of skin cutaneous melanoma (SKCM) is ~ 10 mutation per Mb while that of acute myeloid leukemia (LAML) is less than 1 mutation per Mb. Therefore, we decided to cluster cancer types based on the similarity of log-transformed TMB distribution (Supplementary Fig. 8) so that we check the distribution of log-transformed TMB within each group.

For each cancer type, the density of log-transformed TMB was generated by bin of size 1. Then we used K-means clustering method to group cancer types to 5 clusters based on the similarity of the log-transformed TMB density. In each cluster, the mutation data is aggregated for further analysis.

Cancer survival analysis

Kaplan-Meier survival analysis was used to estimate the association of cancer subtype with the overall survival of patients with colorectal, endometrial, and stomach cancers aggregated data. Furthermore, we performed proportional hazard ratio analysis using the coxph function in R, including cancer, stage, and subtypes as covariates. The significances of the covariates were assessed by Wald tests. Overall survival was calculated from the date of initial diagnosis of cancer to disease-specific death (patients whose vital status is termed dead) and months to last follow-up (for patients who are alive).

Background mutation prediction by BMR model

Within each cancer type, WES data from the training set was used to determine parameters for background mutation model either by using the GLM or the three-steps approach. Background mutations were predicted based on [9] for non-synonymous mutation and [10] for synonymous mutation in both training set and rest of test set.

$$\#\ of\ expected\ background\ non-synonymous\ mutations\ V_{sgn} = \alpha_g b_s E_{g(non-synoymous)} \quad [9]$$

$$\#\ of\ expected\ background\ synonymous\ mutations\ V_{sgs} = \alpha_g b_s E_{g(synoymous)} \quad [10]$$

Driver gene detection statistics

1. Significantly mutated genes (SMG)

The probability of observing at least one non-silent mutations of gene g in sample s is 1-P(Y=0) as [11], where V_{sgn} is expected background non-synonymous mutation from [9], p'_g is gene specific dispersion.

$$P(Y=0) = \frac{1}{\Gamma(1)} \left(\frac{1}{1+V_{sgn}\, p'_g}\right)^{p'^{-1}_g} \qquad [11]$$

We consider significantly mutated genes as those having a high number of non-silent mutations across samples, instead of within a particular sample. Thus, the number of samples with mutated g is counted and Poisson binomial[2] is applied to test whether g is significantly mutated across samples.

2. Potential tumor suppressor gene (TSG)

Tumor suppressor genes contribute to tumorigenesis through inactivation. TSGs are enriched for loss-of-function mutations throughout their gene length[3]. Instead of considering all non-silent mutations, we test if a gene is highly disrupted by loss-of-function mutations (nonsense and frame-shift indels are likely to cause loss-of-function). Similar testing strategy is applied to SMGs. We calculate the probabilities of observing at least one loss-of-function mutation per gene per sample and test whether a gene is significantly enriched for loss-of-function mutations across samples.

3. Potential oncogene (OG)

Unlike tumor suppressors, oncogenes cause tumorigenesis by gain-of-function mechanisms. Activating mutations are generally occur recurrently at the same amino acid positions[3]. For oncogenic activity, we test whether missense mutations are clustered in particular amino acids of the protein. In this calculation we pool mutations from all samples together.

To correct for mutations occurred due to the background mutation rate, we introduce a gene-specific correction factor c_g[12]. The expected number of missense mutations is$\alpha_g b_s E_{g(missense)}$.

$$c_g = \max\left(0, 1 - \frac{expected(missense\ mutation)}{observed(missense\ mutation)}\right) \quad [12]$$

The amino acid positions are obtained for all somatic mutations. In case of multiple proteins from the same gene, the protein with the highest number of somatic mutations is chosen. For the protein, the first step is to use a binomial test to test for each amino acid a, with n is the number of mutations observed in a, N denotes the total number of mutations observed in the protein, and $p_b(missense) = \frac{E_{a(missense)}}{\sum_{all\ a\ of\ the\ protein} E_{a(missense)}}$ denotes the probability of observing a missense mutation at the given amino acid.

$$binom(n * c_g, N * c_g, p = p_a(missense) \quad [13]$$

Significant positions (after multiple hypothesis correction with FDR < 0.05) are grouped into clusters within certain amino acids (e.g. 3). Then the binomial test is re-applied to the identified clusters. The results are stable with respect to the number of amino acids chosen.

4. Combined p-value

P-values from SMG, TSG, and OG analyses are adjusted with multiple hypothesis correction. We also provide combined p-values using Fisher's method. Considering the non-independent nature of the three hypotheses, Fisher's combined p-values are not accurate. We suggest using the ranking instead of the combined p-values.

Reference:

1. Youn, A. & Simon, R. Identifying cancer driver genes in tumor genome sequencing studies. *Bioinformatics* **27,** 175–181 (2011).
2. Hong, Y. On computing the distribution function for the Poisson binomial distribution. *Computational Statistics and Data Analysis* **59,** 41–51 (2013).
3. Vogelstein, B. *et al.* Cancer genome landscapes. *Science* **340,** 1546–1558 (2013).
